# A Pediatric Genetic Disorder Diagnosed in Adulthood

**DOI:** 10.1371/journal.pmed.0030015

**Published:** 2006-01-31

**Authors:** Joseph A Church

**Affiliations:** **1**the Childrens Hospital Los Angeles and Keck School of Medicine, University of Southern California, Los Angeles, United States of America

## Abstract

Church's case report is a reminder that "pediatric" genetic diseases are not limited to children.

## Presentation of Case

A 41-year-old non-Hispanic white woman, accompanied by her psychiatrist, presented for immunologic consultation with an armload of medical records. The patient's primary concerns were environmental mold exposure and a productive cough attributed to recurrent upper and lower respiratory tract infections. Although initially relating the beginning of her infections to mold exposure over the previous four years, the patient had experienced frequent respiratory tract infections since childhood, including recurrent otitis media, mastoiditis, and two episodes of pneumonia. The patient also claimed to have congenital granulocytopenia, specifically Kostmann syndrome.

The patient expressed particular concern about “bowel problems,” which she claimed manifested as “steatorrhea,” inability to gain weight, and a need to eat every three to four hours to stave off emotional agitation. The patient described her stools as “rock hard” to “slimy.” Several gastroenterologists had evaluated the patient over the years, and results of endoscopies and biopsies ruled out celiac disease, chronic infections, and inflammatory bowel disease. The patient provided a list of psychotropic medications prescribed for her “atypical bipolar disorder” that included her specific comments regarding each ( Table 1). At her initial visit, the patient was taking vitamin C supplements, acidophilus, and bovine colostrum. Family history revealed paternal alcohol abuse and kidney stones, and maternal “synthetic chemical sensitivity” and hypoglycemia. No family members had documented intestinal or infectious disorders.

**Table 1:**
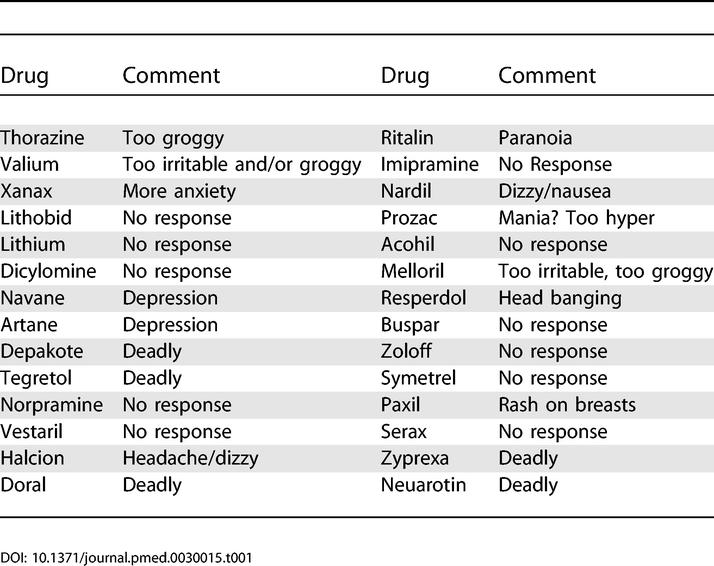
Patient's List of Previously Prescribed Psychotropic Agents and Her Comments Regarding Each

On examination, the patient was well oriented, petite, and anxious. Her height and weight were 158.4 cm and 45.9 kg, respectively. Her blood pressure was 107/64 mm Hg. There was no evidence of active upper or lower respiratory tract infection. Her cardiovascular, abdominal, and lymph node examinations were normal. There were no focal neurologic abnormalities. However, the patient displayed labile emotions and required frequent calming by her psychiatrist.

Laboratory studies performed approximately two years previously revealed the following: hematocrit, 0.42; hemoglobin, 138 g/l; white blood cell count, 2.4 × 10 ^9^/l; absolute neutrophil count (ANC), 0.576 × 10 ^9^ /l; serum IgG, 5.8 g/l (normal = 6.13–12.9 g/l); normal T cell subsets; normal blood chemistry profile; negative antineutrophil antibodies; and negative hepatitis B and C serologies. One year later, the patient's ANC was 0.208 × 10 ^9^/l. Antibody responses to diphtheria and tetanus toxoid vaccines were normal, but antibody responses to 12 pneumococcal serotypes, tested following immunization with unconjugated 23-valent pneumococcal vaccine, were absent. Laboratory studies at initial consultation confirmed neutropenia (ANC, 0.351 × 10 ^9^/l), borderline IgG (6.10 g/l), and absent pneumococcal antibody levels. Pancreatic exocrine insufficiency was demonstrated by a low stool pancreatic elastase level (93 mcg/g; normal is greater than 200 mcg/g).

Despite confounding emotional issues and exaggerated concerns regarding environmental mold exposure, the patient's chronic neutropenia, malabsorption, and subtle immune deficiency were suggestive of a unifying diagnosis: Shwachman-Diamond syndrome (SDS) [ 1]. Genomic analysis was performed by GeneDx, Gaithersburg, Maryland, United States. Two mutations were identified in the patient's DNA sample and confirmed by restriction enzyme analysis of a second DNA amplification. Both mutations mimic the *SBDS* pseudogene and have been seen in numerous patients with SDS [ 2]. The mutations are designated c.183_184 TA to CT, and IVS2+2 T to C. The former mutation causes a chain termination mutation in exon 2 at codon 62, and the latter causes aberrant splicing of the adjacent intron 2. The latter mutation is also known as c.258+2 T to C. The most likely interpretation of this finding is SDS compound heterozygosity, with a different *SBDS* mutation inherited from each parent. The patient has not been seen again at this institution since the diagnosis was made.

## DISCUSSION

For over 40 years, this patient's primary diagnosis was unsuspected. Multiple specialty consultations failed to recognize the importance of complaints affecting organ systems outside the areas of special interest. The patient's psychiatric history and emotional lability complicated assessment, and likely led to the suspicion that her symptoms were psychosomatic.

The attribution of a “weakened immune system” to the presence of home mold contamination led to the patient's referral to a center that deals in both pediatric and adult immune dysfunctions. SDS was suspected, and the diagnosis was confirmed with genetic analysis for *SBDS* gene mutations.

SDS is an autosomal recessive disorder with clinical features that include pancreatic exocrine insufficiency, dysfunctional hematopoiesis, and skeletal abnormalities [ 1, 3]. Cases of SDS with humoral immune deficiency similar to that identified in this patient have been described previously [ 4, 5]. The disorder has been complicated by leukemia [ 6], and described as an inherited pre-leukemic bone marrow failure disorder [ 7]. Until recently, the diagnosis of SDS was based on clinical characteristics in children with growth failure that resembles that of cystic fibrosis [ 1].

Two major internal medicine textbooks [ 8, 9] do not index SDS in the differential diagnosis of either leucopenia or pancreatic exocrine insufficiency, and it is likely that SDS is more common in adults than generally diagnosed.

In 2003, Booncock et al. reported that mutations in a previously uncharacterized gene, *SBDS,* were associated with SDS [ 2]. Recent work demonstrated that the SBDS protein is involved in RNA metabolism [ 10], yet the specific pathways leading from genetic mutations to clinical manifestations have not been delineated. Of particular interest is the finding that SDS-associated bone marrow failure appears to be caused by increased apoptosis associated with hyperactivation of the Fas signaling pathway [ 11]. Perhaps RNA metabolic defects in hematopoetic, skeletal, and exocrine pancreatic progenitors induce up-regulation of Fas expression and programmed cell death in the specific target cells.

The increasing availability of genetic assays, albeit tempered by cost in many circumstances, allows for the definitive diagnosis of genetic conditions, such as SDS, in atypical patients. In the present case, such an assay was particularly useful for identifying a specific pathophysiologic process in a patient with multiple somatic complaints, a complex psychiatric condition, and a genetic “disease of childhood.”

**Learning Points**

• Shwachman-Diamond syndrome should be considered in the differential diagnosis of neutropenia, recurrent respiratory tract infections, and fat malabsorption, and strongly suspected when all three features are present in a single patient.

• “Pediatric” genetic diseases are not limited to children.

• Reliance on clinical descriptions of genetic disorders is likely to lead to underdiagnosis of adult patients with less than classical findings.

• The availability of reliable genetic testing in both academic and commercial settings allows for the definitive diagnosis of diseases previously classified on clinical criteria only.

• A rigorous search for underlying medical conditions may be appropriate even in patients with multiple somatic complaints and psychiatric disorders.

## Abbreviations

ANC: absolute neutrophil count
SDS: Shwachman-Diamond syndrome
